# Cross Sectional Associations between Socio-Demographic Factors and Cognitive Performance in an Older British Population: The European Investigation of Cancer in Norfolk (EPIC-Norfolk) Study

**DOI:** 10.1371/journal.pone.0166779

**Published:** 2016-12-08

**Authors:** Shabina A. Hayat, Robert Luben, Nichola Dalzell, Stephanie Moore, Serena Anuj, Fiona E. Matthews, Nick Wareham, Carol Brayne, Kay-Tee Khaw

**Affiliations:** 1 Department of Public Health and Primary Care, Institute of Public Health, University of Cambridge, Cambridge, United Kingdom; 2 MRC Biostatistics Un it, Cambridge Biomedical Campus, Cambridge Institute of Public Health, Cambridge, United Kingdom; 3 MRC Epidemiology Unit, University of Cambridge School of Clinical Medicine, Cambridge United Kingdom; Seoul National University, REPUBLIC OF KOREA

## Abstract

**Background:**

Cognition covers a range of abilities, such as memory, response time and language, with tests assessing either specific or generic aspects. However differences between measures may be observed within the same individuals.

**Objective:**

To investigate the cross-sectional association of cognitive performance and socio-demographic factors using different assessment tools across a range of abilities in a British cohort study.

**Methods:**

Participants of the European Prospective Investigation of Cancer (EPIC) in Norfolk Study, aged 48–92 years, underwent a cognitive assessment between 2006 and 2011 (piloted between 2004 and 2006) and were investigated over a different domains using a range of cognitive tests.

**Results:**

Cognitive measures were available on 8584 men and women. Though age, sex, education and social class were all independently associated with cognitive performance in multivariable analysis, different associations were observed for different cognitive tests. Increasing age was associated with increased risk of a poor performance score in all of the tests, except for the National Adult Reading Test (NART), an assessment of crystallized intelligence. Compared to women, men were more likely to have had poor performance for verbal episodic memory, Odds Ratio, OR = 1.99 (95% Confidence Interval, 95% CI 1.72, 2.31), attention OR = 1.62, (95% CI 1.39, 1.88) and prospective memory OR = 1.46, (95% CI 1.29, 1.64); however, no sex difference was observed for global cognition, OR = 1.07 (95%CI 0.93, 1.24). The association with education was strongest for NART, and weakest for processing speed.

**Conclusion:**

Age, sex, education and social class were all independently associated with performance on cognitive tests assessing a range of different domains. However, the magnitude of associations of these factors with different cognitive tests differed. The varying relationships seen across different tests may help explain discrepancies in results reported in the current literature, and provides insights into influences on cognitive performance in later life.

## Introduction

Cognitive ability covers a range of domains, which together form the basis of cognitive function. These domains include recall, learning, understanding, encoding and recognition, most of which require prior knowledge and experience. Measuring cognitive abilities is not straightforward because they are not clearly distinct from one another. One ability may have an impact on the performance of another, or different abilities work in conjunction to execute a function, thus making selection of cognitive tests and the interpretation of results difficult.

There is a growing interest in the heterogeneity of cognitive performance observed in the ageing population [1] and what might constitute ‘normal cognitive ageing’ and why decline occurs [2,3]. There are many studies, both longitudinal and cross-sectional investigating the effects of age on cognition or cognitive ageing, however discrepant age trends have been reported where age-related changes observed in cross-sectional studies have not always been observed in longitudinal analyses. It cannot be assumed that results from longitudinal studies will reflect more accurate age related trends than cross-sectional analyses, as selective attrition and practice effects in longitudinal comparisons may under-estimate age-related cognitive decline [2,4].

There is also uncertainty about patterns of decline across different cognitive abilities. Certain abilities such as memory, spatial ability and processing speed have been observed to decline more readily than others, such as comprehension and vocabulary which tend to remain stable for longer [5,6]. Some suggest that decline in mental speed contributes to the decline seen in other abilities [7,8]. Studies have also indicated that decline occurs at global and at domain specific level [9–12] and so both should be tested. As yet, there does not appear to be an agreement on the classification of the cognitive domains and there is a lack of consensus on which abilities are most important in testing cognitive impairment or decline [5].

The difficulties and limitations associated with assessment of cognitive function as a result of the variability in cognitive testing and methodologies restricting cross study comparisons are well known [13,14]. Although a plethora of cognitive assessment tools exist, test performances vary depending on the populations in which they are being used. Low levels of accuracy for detecting mild impairment, demographic biases and a lack of an agreed battery of tests appropriate for use in differing situations, all add to the complexities [13]. It is therefore essential to also consider the psychometric properties of the assessment tool and how well it measures the construct of interest within a particular setting.

To better understand when and where decline begins it is necessary to first identify potential factors for the range of results observed. This includes recognizing the reason for the variability observed between different cognitive tests. The aim of this analysis is to gain further insight into how performance on a range of widely used cognitive assessment tools may relate to the socio-demographic factors; age, sex, social class and education.

## Methods

### Ethics Statement

This research was conducted in compliance with the principles expressed in the Declaration of Helsinki. This study was approved by the Norfolk Local Research Ethics Committee (05/Q0101/191) and East Norfolk and Waveney NHS Research Governance Committee (2005EC07L). Participants provided signed informed consent.

### Participants

Detailed descriptions of recruitment and study methods have been reported elsewhere [15]. Briefly, men and women then aged 40–79 years were recruited at baseline between 1993 and 1997 through registers in thirty-five general practices in Norfolk. The cohort was similar to the national population samples studied in the Health Survey of England, in terms of anthropometry, serum lipids and blood pressure [15]. Such registers function as population based registers in the UK National Health Service. The data presented here are from the third health examination (3HC or EPIC-Norfolk 3), a face to face assessment conducted between 2006 and 2011 including data from the pilot phase between 2004 and 2006 [16] when the participants were aged 48 to 92 years. Although the EPIC-Norfolk 3 (including the pilot) ran over 7 years, the administration of the health examination remained unchanged with staff tightly following a protocol thus minimizing variation, differences in interpretation and reducing subjectivity [17]. Details on the standardized protocol for both test administration and scoring have been described previously [17].

### Assessment of cognition

Cognitive performance was assessed using a battery of tests aiming to cover a range of abilities. These included memory (retrospective and prospective), executive function, attention, language, reading ability, and reaction time, as well as calculation, registration, abstract thinking, learning and visuospatial ability. The test battery took on average 35 minutes to complete.

The cognition battery used in EPIC-Norfolk 3, (EPIC-COG), comprised of seven tests used previously in other studies that are aimed at particular domains of cognitive function. These included testing for i) global cognitive function, using a shortened version of the Extended Mental State Exam (SF-EMSE) [18] a brief pen and paper test; ii) verbal episodic memory, assessed with the Hopkins Verbal Learning Test (HVLT) [19], with words displayed on a screen but requiring only a verbal response; iii) non-verbal episodic memory, using a computerized touch screen test called Cambridge Neuropsychological Test Automated Battery Paired Associates Learning Test (CANTAB-PAL) [20,21], using the first trial memory score (FTMS) as the outcome measure; iv) attention, assessed with a pen and paper letter cancellation task [22] as used in the Medical Research Council Cognitive Function and Ageing Study (MRC CFAS) [23]; using accuracy score (PW-Accuracy), which is the number of correctly identified target letters (Ps and Ws) minus all potential target letters missed up to the point scanned by the participant as the outcome measure; v) prospective memory, an event and time based task (also used in MRC CFAS) [24]); vi) processing speed (reaction time in milliseconds), using the computerized Visual Sensitivity Test (VST) [25,26] and finally vii) reading ability and crystallized intelligence that reflects accumulated knowledge [27] using a shortened version of the National Adult Reading Test [28] or Short-NART [29]. Here we used the NART Error Score as the outcome measure, where a higher score indicates poorer performance. The NART words were presented on a computer screen, but only required a verbal response scored by the researcher as correct or incorrect. The tests as they were used in this population have been described in considerable detail previously [17], and so further details on these tests will not be given here.

**Fig 1:**
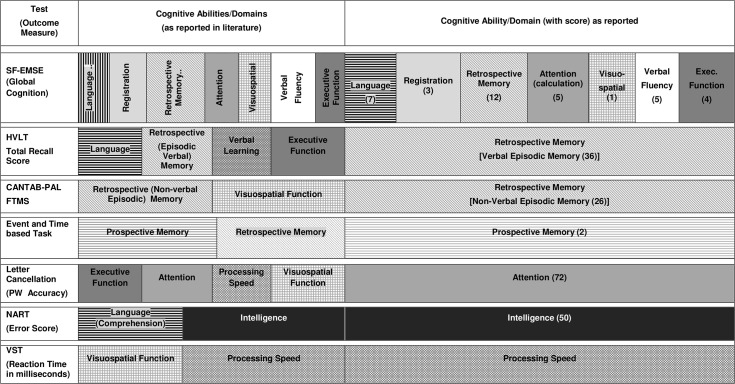
Cognitive abilities assessed by individual test of the EPIC-COG Battery. Cognitive abilities assessed by each test of the EPIC-COG Battery used in the EPC-Norfolk 3 Cohort, 2006–2011 (including data from the pilot phase 2004–2006)

All the above measures of cognitive function were offered to the individuals taking part. Fig 1 shows the range of cognitive abilities purported to be assessed from the literature describing these tests and the predominant ability that is reflected in the score as reported here. Table 1 presents descriptive data showing the characteristics of the men and women in the EPIC-Norfolk cohort.

**Table 1:** Characteristics of the 8584 individuals with cognitive measures participating in EPIC-Norfolk 3, and the pilot phase

	Men		Women	
	(N = 3840)		(N = 4744)	
**Mean Age (SD)**	69.4	(8.1)	68.1	(8.0)
**Frequency % (N)**				
**Age Band**				
<60 years	10.3	(397)	14.0	(665)
60-<65 years	22.7	(871)	26.4	(1252)
65-<70 years	20.9	(802)	20.8	(985)
70-<75 years	19.8	(760)	17.4	(825)
75-<80 years	15.4	(591)	12.9	(613)
80+ years	10.9	(419)	8.5	(404)
**Marital status**				
Single	3.1	(116)	4.0	(184)
Married	86.6	(3244)	71.5	(3314)
Widowed	6.2	(234)	16.3	(754)
Separated or Divorced	4.0	(152)	8.3	(386)
**Education**				
Degree Level	20.1	(772)	15.6	(741)
A level and equivalent	48.0	(1844)	41.1	(1951)
O Level and equivalent	9.8	(377)	13.6	(647)
No Qualifications	22.0	(846)	29.6	(1404)
**Social Class**				
Professional	9.5	(364)	8.2	(384)
Managerial	42.7	(1628)	39.8	(1869)
Skilled Non Manual	12.2	(466)	19.1	(898)
Skilled Manual	22.7	(865)	18.8	(883)
Semi-Skilled Manual	11.0	(418)	11.3	(532)
Non-skilled	1.9	(72)	2.7	(125)
**Mean Cognitive Test Score (SD)**				
SF-EMSE	32.5	(3.2)	32.7	(3.0)
HVLT	23.9	(5.5)	26.1	(5.4)
FTMS	15.2	(4.6)	15.6	(4.5)
PW-Accuracy	11.6	(6.1)	13.1	(6.0)
VST, Rxn Time				
(milliseconds)	2282.5	(430.6)	2229.9	(455.4)
NART Error Score	17.9	(10.3)	16.6	(9.5)
Frequency % (N)				
Prospective Memory Task	77.7 (2912)		84.1 (3915)	
(Successful)				

Abbreviations: A Level, Advanced Level; CANTAB-PAL, Cambridge Neuropsychological Test Automated Battery Paired Associates Learning Test; FTMS, First Trial Memory Score; HVLT, Hopkins Verbal Learning Test; N, Number; NART, National Adult Reading Test; O Level, Ordinary Level; Rxn, Reaction; SF-EMSE:, Shortened version (Short form) of the Extended Mental State Exam; SD, Standard deviation; VST, Visual Sensitivity Test

### Covariates

Age was categorized into 5-year age bands. For marital status, very few participants reported being separated (less than one percent), so this category was combined with the divorced group giving four categories; married, single, widowed and divorced or separated.

Education was obtained from the baseline questionnaire, based on the highest qualification attained, categorized into 4 groups: degree or equivalent, Advanced (‘A’) level or equivalent (educational attainment to the equivalent of completing schooling up to the age of 18 years), Ordinary (‘O’) level or equivalent (a level lower than ‘A’ level, or completing schooling up to the age of 16 years) and finally less than ‘O’-level or no qualifications.

Social class, also from baseline for men was coded using current occupation except if participants reported being unemployed in which case their partner’s social class was used. Last employment was used for men who were retired. Unemployed men without partners were unclassified. Social class in women was graded using the “conventional” approach [30], which uses their partner’s social class except when the partner’s social class was unclassified or missing, or they had no partner in which case social class was based on their own occupation. An unemployed woman without a partner was coded as unclassified. Personal measures for social class of individual’s own occupation were also available.

Social class was classified according to the Registrar General’s occupation-based classification scheme into 5 main categories [31]. Social class I consists of professionals, class II includes managerial and technical occupations, class III is subdivided into non-manual and manual skilled workers (III non-manual and III manual), class IV consists of partly skilled workers, and class V comprises unskilled manual workers. Information on marital status was obtained from the health questionnaire completed near the time of the health examination.

Variables were further grouped as follows: Marital status into Married and Single (combining, single, separated, divorced and widowed categories); Social Class into non manual (combining classes I, II and III non-manual) and manual classes (combining manual classes III manual, IV and V). To give further insight into the effects of lower and higher levels of education, this variable was categorized into three groups; (i) No qualification (less than obtaining an ‘O’ level or equivalent or not completing school up to the age of 16), (ii) Completion of school up to the age of 16 or up to the age of 18 (by combining the two categories of attaining ‘O’ level or equivalent and ‘A’ level or equivalent) and finally (iii) those obtaining an education to graduate level (those with a degree or equivalent) or above. Due to low numbers with poor performance in the highest education group, we used the lowest qualification category as the reference category. However, to allow for a more direct comparison of the odds ratios with the other co-variates, we also used education as a dichotomized variable, comparing no qualification with any qualification (combining ‘O’, ‘A’ and degree level).

### Analyses

In this population where the prevalence of dementia and cognitive impairment using accepted standard diagnostic criteria was very low, we were only able to use poor performance as an indicator, where poor performance was defined (on any test) as obtaining a score less than a cutoff point corresponding to the 10th percentile of the population distribution in each of the seven cognitive tests individually. It was not possible to define a cut-point with 10% of the population distribution for the prospective memory task and as 19% of the population failed the task, this was used as the lower cut-point. Participants were then classified into two groups based on the cut-off for each of the tests.

Obtaining a score less than a cutoff point corresponding to the 10th percentile of the population distribution (or failing the prospective memory task) was first examined by univariate analysis, using the chi-square test to observe differences between the groups for each socio-demographic variable. The associations between each variable and being in the poor performance group were assessed using logistic regression analysis adjusting for co-variates; age (per 5 years), sex (women being the reference group), marital status (being married as the reference group), educational level (no qualification being the reference group for the three level education variable, and with qualifications being the reference group for the dichotomized education variable) and social class (non-manual, as the reference group) where social class was examined using both the “conventional” method using a woman’s partner’s occupation, as well as personal measures according to a woman’s own occupation. Data was further examined stratified into two age groups split at age 65 years (creating two groups, those under age 65 and those 65 years and older).

Age was also included in a model as a continuous variable and the unstandardized (Beta) coefficient were examined to compare differences in terms of chronological years of age for education level (comparing those with no qualifications with those completing school to the age of 16 or 18 and secondly, those with no qualifications with those educated to graduate level).

In addition, we included a further model, adding the interaction term age group (< 65 and those ≥65 years) x education (entered as the dichotomized variable, no qualification compared to those with any qualification) to examine difference in poor cognitive performance for age depending on education level. Tests showing a significant interaction between age group and education (no qualification compared to those with any qualification), were then stratified to examine the magnitude of differences in associations for these two age groups.

Although intelligence and education, are said to be correlated [27], we did not assume our tests of crystalized intelligence, NART, to be measuring the same exposure and so we tested this further as a secondary analysis and associations were examined by further adjusting for the NART Error Score.

### Missing data in cognitive tests

To explore the effect of missing data, a sensitivity analysis was carried out only in individuals with complete data on all seven cognitive tests and the specified covariates (n = 5727). All *P* values reported are 2 sided. Statistical analysis was performed using SPSS version 21.0 (IBM Corp., Armonk, NY, USA).

## Results

**Fig 2:**
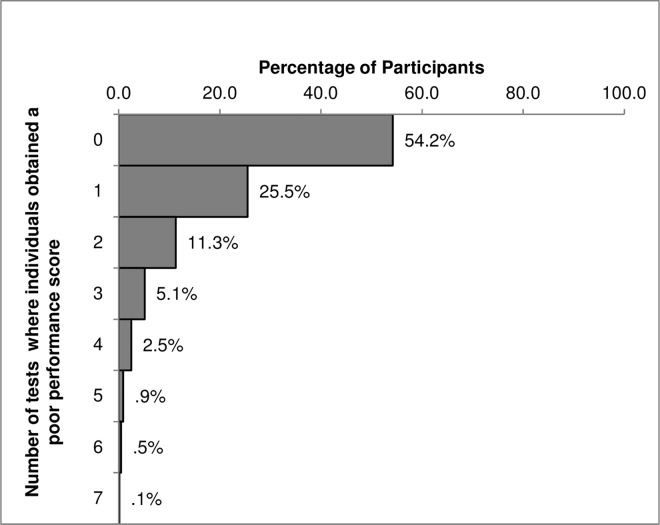
Distribution of participants in the poor performance group across all tests in the EPIC-COG Battery. Distribution of participants in the poor performance group (defined as obtaining a score less than a cut-off point corresponding to the 10th percentile of the population distribution in each of the seven cognitive tests individually.

Of the 8623 participants attending the third examination, cognitive data were available on 8584 men and women. Compliance was high with over 90% (n = 7773) attempting at least six of the seven tests in the battery and almost 70% (n = 6011) having measures for all seven tests. Less than 5% (n = 223) of participants attempted fewer than four tests. The CANTAB-PAL and VST had lower completion rates than the other tests, partly due to a technical computer failure, which resulted in loss of data. Fig 2 shows the distribution of the EPIC-Norfolk participants with poor cognitive performance across the seven tests. Over 50% of participants in the cohort were not in the poor performing group for any test and less than 10% of the cohort had a poor performance score in three or more tests.

The distribution of poor cognitive performance by the socio-demographic variable (age, sex, marital status, education and social class) in each of the tests are shown in Tables 2, 3 and 4.

**Table 2:** Distribution of participants in the bottom tenth percentile in each cognitive test by age.

	SF-EMSE		HVLT		FTMS		PW_Acc		Prospective Memory		VST		NART Error Score	
	Freq.		Freq.		Freq.		Freq.		Freq.		Freq.		Freq.	
	N	N (%)	N	N (%)	N	N (%)	N	N (%)	N	N (%)	N	N (%)	N	N (%)
		Score< = 29	Total	Score< = 18	Total	Score< = 10	Total	Score< = 5	Total	Failed task	Total	> = 2702.46 ms	Total	Score> = 31
All	8483	1092 (12.9)	8081	1042 (12.9)	7281	870 (11.9)	8410	915 (10.9)	8403	1576 (18.8)	7144	714 (10.0)	8112	849 (10.5)
**Age**														
<60														
years	1055	49 (4.6)	1022	30 (2.9)	959	39 (4.1)	1048	51 (4.9)	1049	92 (8.8)	890	59 (6.6)	999	80 (8.0)
60-<65														
years	2105	159 (7.6)	2061	118 (5.7)	1877	125 (6.7)	2098	125 (6.0)	2095	246 (11.7)	1846	112 (6.1)	2062	196 (9.5)
65-<70														
years	1767	200 (11.3)	1700	146 (8.6)	1529	136 (8.9)	1756	169 (9.6)	1751	286 (16.3)	1522	124 (8.1)	1716	201 (11.7)
70-<75														
years	1572	221 (14.1)	1496	263 (17.6)	1339	190 (14.2)	1563	181 (11.6)	1558	336 (21.6)	1323	128 (9.7)	1508	180 (11.9)
75-<80														
years	1183	244 (20.6)	1109	257 (23.2)	985	207 (21.0)	1170	203 (17.4)	1170	325 (27.8)	996	142 (14.3)	1107	131 (11.8)
80+														
years	801	219 (27.3)	693	228 (32.9)	592	173 (29.2)	775	186 (24.0)	780	291 (37.3)	567	149 (26.3)	720	61 (8.5)
p<0.001			p<0.001		p<0.001		p<0.001		p<0.001		p = 0.001		p = 0.001	

P-value for trends across the groups using the chi-square test

Abbreviations: A Level, Advanced Level; FTMS, Freq, Frequency; First Trial Memory Score (outcome measure for CANTAB-PAL, Cambridge Neuropsychological Test Automated Battery Paired Associates Learning Test); HVLT, Hopkins Verbal Learning Test; Ms, millisecond; NART, National Adult Reading Test; N, Number; O Level, Ordinary Level; SF-EMSE:, PW-Accuracy (Accuracy Score, for the letter cancellation task) Shortened version (Short form) of the Extended Mental State Exam; VST, Visual Sensitivity Test

**Table 3:** Distribution of participants in the bottom tenth percentile in each cognitive test by sex and marital status.

	SF-EMSE		HVLT		FTMS		PW_Acc		Prospective Memory		Visual sensitivity		NART Error Score	
	Freq		Freq.		Freq.		Freq.		Freq.		Freq.		Freq.	
	N	N (%)	N	N (%)	N	N (%)	N	N (%)	N	N (%)	N	N (%)	N	N (%)
		Score< = 29	Total	Score< = 18	Total	Score< = 10	Total	Score< = 5	Total	Failed task	Total	> = 2702.46 ms	Total	Score> = 31
**Sex**														
Women	4681	580 (12.4)	4476	424 (9.5)	4011	442 (11.0)	4650	409 (8.8)	4653	738 (15.9)	3928	364 (9.3)	4504	397 (8.8)
Men	3802	512 (13.5)	3605	618 (17.1)	3270	428 (13.1)	3760	506 (13.5)	3750	838 (22.3)	3216	350 (10.9)	3608	452 (12.5)
	p = 0.14		p<0.001		p = 0.007		p<0.001		p<0.001		p = 0.02		p<0.001	
**Marital**														
**Status**														
Single	295	42 (14.2)	282	35 (12.4)	251	50 (19.9)	291	35 (12.0)	239	54 (18.4)	241	28 (11.6)	283	27 (9.5)
Married	6494	777 (12.0)	6225	766 (12.3)	5620	597 (10.6)	6445	638 (9.9)	6439	1149 (17.8)	5499	521 (9.5)	6244	656 (10.5)
Widowed	964	173 (17.9)	883	165 (18.7)	786	150 (19.1)	948	163 (17.2)	948	244 (25.7)	779	112 (14.4)	895	93 (10.4)
Separated														
or divorced	533	68 (12.8)	499	43 (8.6)	445	37 (8.3)	530	53 (10.0)	529	86 (16.3)	450	34 (7.6)	503	40 (8.0)
	p<0.001		p<0.001		p<0.001		p<0.001		p<0.001		p<0.001		p = 0.3	

P-value for trends across the groups using the chi-square test

Abbreviations: A Level, Advanced Level; Freq, Frequency, FTMS, First Trial Memory Score (outcome measure for CANTAB-PAL, Cambridge Neuropsychological Test Automated Battery Paired Associates Learning Test); HVLT, Hopkins Verbal Learning Test; Ms, millisecond; NART, National Adult Reading Test; N, Number; O Level, Ordinary Level; SF-EMSE:, PW-Accuracy (Accuracy Score, for the letter cancellation task) Shortened version (Short form) of the Extended Mental State Exam; VST, Visual Sensitivity Test

**Table 4:** Distribution of participants in the bottom tenth percentile in each cognitive test by social class and education

	SF-EMSE		HVLT		FTMS		PW_Acc		Prospective Memory		VST		NART Error Score	
	Freq		Freq		Freq		Freq		Freq		Freq		Freq	
	N	N (%)	N	N (%)	N	N (%)	N	N (%)	N	N (%)	N	N (%)	N	N (%)
	(Total)	Score< = 29	(Total)	Score< = 18	(Total)	Score< = 10	(Total)	Score< = 5	(Total)	Failed task	(Total)	> = 2702.46 ms	(Total)	Score> = 31
**Social Class**														
Professional	734	58 (7.9)	694	59 (8.5)	649	55 (8.5)	728	55 (7.6)	727	102 (14.0)	646	58 (9.0)	709	15 (2.1)
Managerial	3445	342 (9.9)	3306	336(10.2)	2957	316 (10.7)	3416	312 (9.1)	3416	603 (17.7)	2893	266 (9.2)	3311	188 (5.7)
Skilled														
Non Manual	1350	149 (11.0)	1286	168 (13.1)	1139	140 (12.3)	1342	142 (10.6)	1342	233 (17.4)	1145	114 (10.0)	1301	92 (7.1)
Manual	1736	291 (16.8)	1644	267 (16.2)	1493	187 (12.5)	1722	228 (13.2)	1715	377 (22.0)	1453	148 (10.2)	1633	332 (20.3)
Semi-skilled														
Manual	943	192 (20.4)	893	163 (18.3)	811	132 (16.3)	932	130 (13.9)	932	203 (21.8)	782	90 (11.5)	900	161 (17.9)
Non-skilled	196	48 (24.5)	185	41 (22.2)	165	30 (18.2)	193	30 (15.5)	194	41 (21.1)	157	24 (15.3)	178	51 (28.7)
	p<0.001		p<0.001		p<0.001		p<0.001		p<0.001		p = 0.08		p<0.001	
**Education**														
Degree Level	1491	73 (4.9)	1415	71 (5.0)	1283	86 (6.7)	1484	101 (6.8)	1484	196 (13.2)	1264	97 (7.7)	1447	15 (1.0)
A level														
and equiv.	3749	429 (11.4)	3596	449 (12.5)	3233	352 (10.9)	3719	412 (11.1)	3720	668 (18.0)	3195	295 (9.2)	3604	289 (8.0)
O Level														
and equiv.	1019	76 (7.5)	976	77 (7.9)	895	87 (9.7)	1013	60 (5.9)	1011	163 (16.1)	870	74 (8.5)	966	42 (4.3)
No														
Qualifications	2222	512 (23.0)	2092	443 (21.2)	1868	344 (18.4)	2192	341 (15.6)	2186	547 (25.0)	1813	248 (13.7)		2093	502 (24.0)
	p<0.001		p<0.001		p<0.001		p<0.001		p<0.001		p<0.001		p<0.001	

P-value for trends across the groups using the chi-square test

Abbreviations: A Level, Advanced Level; Freq, Frequency; FTMS, First Trial Memory Score (outcome measure for CANTAB-PAL, Cambridge Neuropsychological Test Automated Battery Paired Associates Learning Test); eqiv, equivalent; HVLT, Hopkins Verbal Learning Test; Ms, millisecond; NART, National Adult Reading Test; N, Number; O Level, Ordinary Level; SF-EMSE:, PW-Accuracy (Accuracy Score, for the letter cancellation task) Shortened version (Short form) of the Extended Mental State Exam; VST, Visual Sensitivity Test

Associations adjusted for the covariates age, sex, education and social class are shown in Table 5 (associations adjusted for age only are given in S1 Table).

**Table 5:** Odds ratios for poor performance adjusted for age, sex, marital status, education and social class.

	SF-EMSE		HVLT		FTMS		PW-Accuracy		Prospective Memory		VST		NART Error Score	
	OR (95% CI)		OR (95% CI)		OR (95% CI)		OR (95% CI)		OR (95% CI)		OR (95% CI)		OR (95% CI)	
Number of Participants	8208		7817		7036		8138		8403		6902		7846	
**Age**	1.43	1.37, 1.50	1.68	1.60, 1.77	1.5	1.42, 1.58	1.38	1.32, 1.45	1.38	1.33, 1.43	1.37	1.30, 1.45	0.96	0.91, 1.007
(per 5 year increase)	p<0.001		p<0.001		p<0.001		p<0.001		p<0.001		p<0.001		p = 0.09	
**Sex**	1.07	0.93, 1.24	1.99	1.72, 2.31	1.17	1.00, 1.36	1.62	1.39, 1.88	1.46	1.29, 1.64	1.16	0.98, 1.37	1.77	1.51, 2.08
(Women ^a^)	0.3		p<0.001		p = 0.06		p<0.001		p<0.001		p = 0.08		p<0.001	
**Marital status**	1.08	0.92, 1.28	0.99	0.83,1.19	1.26	1.05, 1.50	1.35	1.14, 1.61	1.11	0.96, 1.27	1.04	0.85, 1.27	1.04	0.85, 1.27
(Married ^a^)	p = 0.3		p = 0.9		p = 0.01		p = 0.001		p = 0.2		p = 0.7		p = 0.7	
**Social Class**	1.68	1.45, 1.94	1.52	1.31, 1.77	1.24	1.06, 1.46	1.42	1.22, 1.66	1.25	1.10, 1.42	1.1	0.92, 1.31	2.63	2.23, 3.09
(Non-manual ^a^)	p<0.001		p<0.001		p = 0.01		p<0.001		p = 0.001		p = 0.3		p<0.001	
**Education**														
Up to16 or 18 years	0.5	0.43,0.58	0.59	0.51, 0.69	0.65	0.55,0.77	0.72	0.62, 0.85	0.74	0.65, 0.84	0.74	0.62,0.89	0.27	0.23, 0.32
(No Qual ^a^)	p<0.001		p<0.001		p<0.001		p<0.001		p<0.001		p = 0.002		p<0.001	
Graduate Level	0.25	0.19, 0.33	0.26	0.20, 0.35	0.43	0.33,0.57	0.54	0.41, 0.69	0.56	0.46, 0.69	0.67	0.51, 0.88	0.05	0.003,0.008
(No Qual ^a^)	p<0.001		p<0.001		p = 0.001		p<0.001		p<0.001		p = 0.004		p<0.001	

^a^ Reference category

CI, Confidence Interval; FTMS, First Trial Memory Score (outcome measure for CANTAB-PAL, Cambridge Neuropsychological Test Automated Battery Paired Associates Learning Test); HVLT, Hopkins Verbal Learning Test; N, Number; NART, National Adult Reading Test; OR, Odds ratio; PW-Accuracy (Accuracy Score, for the letter cancellation task) Qual, Qualifications; SF-EMSE, Shortened version (Short form) of the Extended Mental State Exam; SD, Standard deviation, VST, Visual Sensitivity Test

Odds Ratios for poor performance (defined as obtaining a score less than a cut-off point corresponding to the 10th Percentile of the population distribution) for each test in the cognition battery used in EPIC-Norfolk 3, adjusted for covariates (age, sex, marital Status, education and social class)

### Age

Table 5 indicates that increasing age was associated with being in the poor performance group for all tests, except the NART error score (intelligence) for which there was no significant trend with age in the multivariable model, Odds Ratio (OR) = 0.96 (95% Confidence Interval (CI) 0.91, 1.01 *P* = 0.09). The strongest association was observed for HVLT OR = 1.68 (95% CI 1.60, 1.77 *P*<0.001) followed by FTMS OR = 1.50 (95% CI 1.42, 1.58 *P*<0.001), both testing for episodic memory. The OR observed for the tests for the other domains were more comparable to each other.

#### Stratifying data by age group (<65 and ≥65 years of age)

When the data were stratified into the two age groups (<65 years and ≥65 years), the most striking difference in association between the age groups was observed for the VST for both age and sex, where for the under 65 group, there was no association between age and poor performance, OR = 0.9 (95% CI 0.75, 1.15 P = 0.5) or for sex, OR = 0.99 (95% CI 0.71, 1.37 P = 0.9), but statistically significant associations observed in the 65+ years age group for age, OR = 1.52 (95% CI 1.40, 1.66 P<0.001) and sex OR = 1.23 (95% CI 1.01, 1.50 P = 0.04). The results for the stratified analyses are shown in S2 Table.

### Sex

There were noticeable differences in the odds ratios for gender across the different tests. Men were more likely to be in the poor performance group for HVLT, PW-Accuracy, the prospective memory task and NART. Weaker, but not statistically significant associations were observed for FTMS and VST. There were no differences observed between men and women for SF-EMSE.

### Marital status

Marital status was significantly associated with cognitive performance for FTMS and PW-Accuracy, with poor performance more likely in single than married individuals. The other tests showed no evidence of associations with marital status.

### Education

More education was strongly associated with decreased poor performance across all the tests. However, the strength of association varied from one test to another, with NART being most strongly associated, particularly at graduate level.

When education was examined as a dichotomized variable (so that the ORs for education were directly comparable with the other covariates), this time comparing no qualification with any qualification (combining ‘O’, ‘A’ and degree level), and using the qualification group as the reference, the OR observed were; SF-EMSE OR = 2.22 (95% CI 1.92, 2.56 *P*<0.001); HVLT OR = 1.89 (95% CI 1.62, 2.20 *P*<0.001); FTMS OR = 1.63 (95% CI 1.38, 1.93 *P*<0.001); Prospective memory OR = 1.42 (95% CI 1.24, 1.62 *P*<0.001); PW-Accuracy OR = 1.45 (95% CI 1.23, 1.70 *P*<0.001); VST OR = 1.37 (95% CI 1.14, 1.65 *P =* 0.001) and NART 4.31 (95% CI 3.66, 5.08 *P*<0.001). Again, any qualification compared to no qualification having the strongest association for the NART (data not shown).

Using the unstandardized regression coefficients (from the model adjusting for covariates in Table 5, unstandardized coefficients not shown), we observed that the likelihood of being in the poor performance group for those with an education up to ‘O’ or ‘A’ level compared to no qualification was equivalent to being just over 5 years younger for HVLT, FTMS, PW-Accuracy, prospective memory task, and VST. Comparing graduate level to no qualification, the likelihood was equivalent to being almost 10 years younger for most of the tests and 13 years younger for the HVLT. For the SF-EMSE, comparing those with an education up to ‘O’ or ‘A’ level compared to those with no qualification, was equivalent to 9.9 years younger and for graduate level, compared to no qualifications the likelihood was equivalent to those 19.6 years younger (data not shown).

There was significant interaction between education (no qualification compared to those with any qualification) for HVLT (p = 0.01) and FTMS (p = 0.01) but not for SF-EMSE, PW_Accuracy, prospective memory, VST or NART. On stratifying the data for both HVLT and FTMS (complete data not shown), the associations were stronger with education for the under 65 age group for HVLT, OR = 3.42 (95% CI 2.35, 4.99 P<0.001) than for the ≥65 year age group OR = 1.94 (95% CI 1.65, 2.28 P<0.001); and for FTMS, the under 65 age group OR = 2.50 (95% CI 1.73, 3.62 P<0.001) and ≥65 year age group OR = 1.66 (95% CI 1.39, 1.99 P<0.001). This can be seen in the associations observed for education in the age group stratified data (S2 Table), particularly for education to 16 or 18 years for HVLT and FTMS. Associations were considerably stronger for the <65 years age group as compared to the ≥65 years for both these tests. The association with education did not differ greatly in the two age groups for the remaining tests.

### Social Class

Manual social class was independently associated with poor performance for all the tests apart from VST. On repeating the model but using individuals’ own social class or occupation rather than the ‘conventional’ method, the associations observed were slightly stronger, however, there were no qualitative differences observed in the relationship. The data for both measures of social class are shown in S3 Table.

### Adjustment for NART

When the models were additionally adjusted for the NART error score as a secondary analysis (S4 Table), the odds were attenuated, but a higher likelihood of poor performance per 5 year increase in age was still observed for all remaining tests. The relationship observed for SF-EMSE reversed, with men 27% less likely to be in the poor performance group than women OR = 0.83, (95% CI 0.71, 0.97) *P* = 0.02. Little change seen was observed in the associations for any of the tests for marital status.

Associations with education at both ‘O’ or ‘A’ level and at graduate level were attenuated but still observed for SF-EMSE and FTMS after adjusting for NART. Graduate level education remained inversely associated with for poor cognitive performance. Associations were no longer significant for education and PW-Accuracy or the prospective memory task, For HVLT, associations with education were observed at graduate level but not at ‘O’ and ‘A’ level. For VST, associations were only observed for those completing school to ‘O’ and ‘A’ level but did not remain at graduate level. For social class, additionally adjusting for NART substantially attenuated associations, with just SF-EMSE still statistically significant association.

### Missing data in cognitive tests

The results from the sensitivity analysis carried out only on individuals with complete data on all tests were similar to earlier results presented. The sensitivity analyses are shown in S5 and S6 Tables.

## Discussion

This paper presents the results of the association of age, sex, education, social class and (as a secondary analysis), crystallized intelligence on different cognitive abilities using a range of assessment tools in a healthy population of older men and women. We observed that the relationship of sociodemographic factors with cognitive function does depend on the assessment tool used.

Older age was found to be associated with poor performance in all of the cognitive tests (abilities), except with the NART score (crystallized intelligence), confirming in this larger study that age has little or no association with NART performance [32]. The greatest age-related differences were observed for HVLT (verbal episodic memory). Episodic memory deficits have been reported to be associated with strongest and most persistent risk of cognitive decline [33,34] and are the most common and earliest complaints. Our findings are consistent with previous work, here presented in terms of poorer performance observed in multiple domains across age [6,11,35] and stability observed in crystallized intelligence, or knowledge that is learned and acquired over years which has shown to be more resistant to the effect of age [28,36,37].

Men were significantly more likely to be poor performers compared to women for HVLT, PW-Accuracy (attention), prospective memory and NART. The sex differences were greatest for HVLT. In contrast, there was no significant sex difference for SF-EMSE (global function). Differences in men and women have been shown across various domains [38], and our findings, were consistent with other studies [39]. Dementia can be considered as the extreme of poor cognitive performance. Though the overall prevalence of dementia has been reported to be higher in women [40] this could be explained by a higher proportion of older women in the general population. Findings from large population-based studies report no sex differences in the rates of dementia up to high age[41–43] with higher rates only observed in women compared to men in the oldest old [43]. The higher prevalence of dementia observed in women may be due to the fact that age is the strongest risk factor for dementia and women make up the majority of the older population due to their increased life expectancy relative to men. No significant differences between men and women were observed for VST (processing speed), although previous studies have found men to perform better [44]. This could be partly explained in the composition of the test which relies on the accurate recognition of a triangle shape as well as speed. Hence accuracy (performed better by women) and reaction time or processing speed (performed better by men), when combined, result in men and women shown to be performing similarly.

As shown previously, [45] education and social class were independently associated with cognitive performance. This was observed across all the domains even when adjusting for age and the other covariates. Although associations were strengthened when assigning social class to women using their own social class, we found no qualitative differences in associations from this and the ‘conventional’ method and the prediction of poor performance in all the tests.

When estimating the potential impact of education on the age-related likelihood of poor performance, this was noticeable for the SF-EMSE test for global cognition, where the risk of being in the poor performance group for those with an education up to ‘O’ and ‘A’ level compared to no qualification was equivalent to being almost 10 years younger and at graduate level compared to no qualification, this difference was even greater, with the risk equivalent to being almost 20 years younger.

We also found in this study that the Influence of education was stronger in younger age group (<65 years) than in ≥65 year age group. Not having qualifications in those <65 years was associated with greater risk of being in the poor performance group compared to people aged 65 and over. Plausible explanations for this difference include the likelihood that other factors such as co morbidities common in older people could have a greater influence on cognitive performance in older people.

NART, as would be expected, showed the greatest association with education, however when controlling for the NART, associations were substantially attenuated but still observed for the SF-EMSE and FTMS for the levels of education examined. The change of direction of the association observed for SF-EMSE and sex after adjusting for NART may be because men performed worse than women on the NART and this was observed in the change in association on adjusting for NART.

Education was associated with a lowered risk of poor performance for HVLT at graduate level but not at ‘O’ and ‘A’ level in the fully adjusted model. Associations were no longer present for PW-Accuracy and little for prospective memory. Education and Intelligence (of which NART is a proxy measure), even though known closely related variables, are not perfectly correlated, and cannot be substituted for each other [27]. Our findings support this and indicate that whatever NART assesses, whether it is a surrogate for prior ability including childhood intelligence or a composite indicator of education; adjusting for NART has a material influence on the independent association of social class and education on cognitive performance measures. Though NART does not completely remove association in all the tests here, it is an important exposure to consider and adjust for when analyzing cognitive function.

Another noteworthy point is that tests purporting to measure similar domains, such as FTMS and HVLT; both measures of episodic memory (one verbal and one non-verbal), showed quite different relationships, particularly for sex, with men showing greater likelihood of poor performance for HVLT than the FTMS. One possible explanation is that tasks requiring verbal and semantic knowledge are considered to be more cognitively demanding, requiring greater self-initiation and encoding than non-verbal tasks such as the FTMS of the CANTAB-PAL [11,46]. This observation, as well as that seen for processing speed, highlights the complex nature for assessing cognitive function, because different abilities do not work in isolation. Each test actually assesses more than the one ability (as shown in Fig 1) and to execute a task successfully, abilities work in conjunction and not independently of each other. While the score focuses on the performance of a single ‘main’ ability, it actually reflects a range of other abilities that are being utilized.

There are a number of strengths to this study. The data are collected from a single large population with individuals being assessed under the same conditions. This reduces the variation in methodology seen in other studies that have combined data from various sources. We also examined ability separately across a wider range covering six domains including a test for global function and not restricted to a few domains, just episodic memory or simply global cognitive function as in many studies. Furthermore, we assessed differences in performance at individual test level rather than as aggregated test scores which allowed us to observe differences even in those tests intending to measure the same ability.

Our cohort is of course confined those with capacity to consent and those willing to participate in the follow up examination, so it is unlikely to include those with major ill health including established dementia. However unlike other studies that are more restrictive in age, sex or using a cohort that is not representative of the general population [3,23,45], our cohort comprises a large number of participants from the general population at baseline, includes men and women, covers a broad range in age, education and social class. Even in this relatively well-functioning group [16], we observed variability in function across all domains. While cross-sectional studies cannot look at longitudinal change, the recruitment bias are similar to longitudinal studies, with healthier, younger individuals more likely to take part. Although longitudinal studies have the advantage of allowing temporal relationship to be examined more accurately, they still have the issue of attrition and drop out which if uncorrected reduces the observed decline.

As mentioned above, a limitation of our study is that we have not tested for other risk factors for poor performance in particular comorbidities, all which influence cognitive performance. These will be examined in further detail in the future as the main focus of this paper was on differences across assessment tools. This study is cross-sectional, and reflects a more ‘healthy profile’ as it consists of survivors and those who make it to the health check and get tested. There are plans to add longitudinal measures to examine the pattern of age related changes in this cohort.

In summary, we observed that the likelihood of poorer cognitive performance increases with age for all measures, in particular for HVLT (verbal episodic memory) but not for NART which is reported to measure crystallized intelligence, or accumulated knowledge. Men were more likely to have poor performance than women in all tests apart from SF-EMSE (global cognition) and marital status having only moderate influence on the FTMS (non-verbal episodic memory) and PW_Accuracy (attention) but not on the other tests. Social class was shown to have the strongest association with the NART (crystallized intelligence) and least with VST (processing speed) and education was associated with performance across all tests, again with the strongest for the NART In conclusion, we report that age, sex, education, social class and crystallized intelligence though all independently related to cognitive performance, varied in their association with the different tests. To further investigate “true” age related changes, characteristics of tests must also be considered when investigating determinants of cognitive function.

While the relationship between cognitive performance and the functioning of an individual or health outcomes is beyond the scope of this paper, these results provide further understanding of the influences and characteristics of these tests. This may enable greater insight into the determinants of cognitive function in older people and how to maintain cognitive health in later life.

## Supporting Information

S1 Table Age adjusted odds ratios for poor performance for each test in the EPIC-COG battery (DOCX)

S2 Table Odds ratios for poor performance stratified by those over and under 65 years. (DOCX)

S3 Table Odds ratios for poor performance using two separate measures of social class for women. (DOCX)

S4 Table Odds ratios for poor performance adjusted for all covariates plus NART Error Score (DOCX)

S5 Table Odds ratios for poor performance for participants with complete data (N = 5727) (DOCX)

S6 Table Odds ratios for poor performance for participants with complete data also adjust for NART. (DOCX)
