# Supplementary material for: Cross Sectional Associations between Socio-Demographic Factors and Cognitive Performance in an Older British Population: The European Investigation of Cancer in Norfolk (EPIC-Norfolk) Study
Source: PLoS One. 2016 Dec 8;11(12):e0166779. doi: 10.1371/journal.pone.0166779 (PMC5145160; doi:10.1371/journal.pone.0166779)
Supplement: S1 Table — (DOCX) [file pone.0166779.s001.docx]

Table S1. Age adjusted odds ratios for poor performance for each test in the EPIC-COG battery

|  | **SF-EMSE**  **Global function** | | | **HVLT**  **Verbal episodic memory** | | | **FTMS**  **Non-verbal episodic memory** | | **PW-Accuracy** | | | **Prospective Memory** | | | **VST Rxn Time**  **Processing Speed** | | **NART Error Score**  **Intelligence** | |
| --- | --- | --- | --- | --- | --- | --- | --- | --- | --- | --- | --- | --- | --- | --- | --- | --- | --- | --- |
|  | OR | | 95% CI  (p value) | OR | | 95% CI  (p value) | OR | 95% CI  (p value) | OR | | 95% CI  (p value) | OR | | 95% CI  (p value) | OR | 95% CI  (p value) | OR | 95% CI  (p value) |
|  |  | | |  | | |  | |  | | |  | | |  | |  | |
|  |  |  | |  |  | |  |  |  |  | |  |  | |  |  |  |  |
| Sex (Men vs Women ^a^) | 1.00 | 0.88-1.14 (*P*=1.00) | | 1.86 | 1.62-2.13 (*P*<0.001) | | 1.09 | 0.94-1.26 (*P*=0.3) | 1.49 | 1.30-1.72 (*P*<0.001) | | 1.42 | 1.27-1.59 (*P*<0.001) | | 1.10 | 0.94-1.29 (*P*=0.2) | 1.47 | 1.28-1.70 (*P*<0.001) |
|  |  |  | |  |  | |  |  |  |  | |  |  | |  |  |  |  |
| Marital status (Single vs Married ^a^) | 1.03 | 0.89-1.21 (*P*=0.7) | | 0.81 | 0.69-0.96 (*P*=0.01) | | 1.19 | 1.00-1.41 (*P*=0.05) | 1.17 | 0.99-1.37 (*P*=0.07) | | 0.99 | '0.86-1.13  (*P*=0.9) | | '1.01 | 0.84-1.22 (*P*=0.9) | 0.87 | '0.72-1.04  (*P*=0.1) |
| Social Class (Manual vs Non- Manual ^a^) | 2.25 | 1.97-2.57 (*P*<0.001) | | 2.04 | 1.77-2.34 (*P*<0.001) | | 1.48 | 1.27-1.72 (*P*=0.01) | 1.65 | 1.43-1.90 (*P*<0.001) | | 1.44 | 1.28-1.62 (*P*<0.001) | | 1.24 | 1.05-1.46 (*P*=0.01) | 4.3 | 3.70-5.00 (*P*<0.001) |
| Education  Up to 16 or 18 vs No Qualifications ^a^ | 0.46 | 0.40, 0.52 (*P*<0.001) | | 0.59 | 0.51, 0.69  (*P*<0.001) | | 0.62 | 0.53, 0.73 (*P*<0.001) | '0.70 | 060, 0.82 (*P*<0.001) | | 0.74 | 0.65, 0.84  (*P*<0.001) | | 0.74 | 0.62, 0.89  (*P*=0.002) | 0.72 | '0.61,0.86  (*P*<0.001) |
| Graduate level vs No Qualifications ^a^ | 0.20 | 0.16, 0.26  (*P*<0.001) | | 0.24 | 0.19, 0.32 (*P*<0.001) | | 0.39 | 0.30, 0.51  (*P*=0.001) | 0.48 | 0.38, 0.61 (*P*<0.001) | | 0.55 | 0.46, 0.66 (*P*<0.001) | | 0.64 | 0.50, 0.82 (*P<0.001*) | 0.03 | 0.02, 0.05 (*P*<0.001) |
|  |  |  | |  |  | |  |  |  |  | |  |  | |  |  |  |  |

^a^ Reference category

Odds Ratios for poor performance (defined as obtaining a score less than a cut-off point corresponding to the 10th Percentile of the population distribution) for each test in the cognition battery used in EPIC-Norfolk 3, adjusted for age only.

Abbreviations: A Level, Advanced Level; CANTAB-PAL, Cambridge Neuropsychological Test Automated Battery Paired Associates Learning Test; CI, Confidence Interval, FTMS, First Trial Memory Score; HVLT, Hopkins Verbal Learning Test; NART, National Adult Reading Test; N, Number; O Level, Ordinary Level; OR, Odds ratio, Rxn, Reaction time; SF-EMSE:, Shortened version (Short form) of the Extended Mental State Exam; SD, Standard deviation; VST, Visual Sensitivity Test
