# Supplementary material for: Cross Sectional Associations between Socio-Demographic Factors and Cognitive Performance in an Older British Population: The European Investigation of Cancer in Norfolk (EPIC-Norfolk) Study
Source: PLoS One. 2016 Dec 8;11(12):e0166779. doi: 10.1371/journal.pone.0166779 (PMC5145160; doi:10.1371/journal.pone.0166779)
Supplement: S2 Table — (DOCX) [file pone.0166779.s002.docx]

Table S2: Odds ratios for poor performance stratified by those over and under 65 years.

|  | **SF-EMSE**  **Global function** | | | | **HVLT**  **Verbal episodic memory** | | **FTMS**  **Non-verbal episodic memory** | | | **PW-Accuracy**  **Attention** | | | | | **Prospective Memory** | | | | | **VST (Rxn Time)**  **Processing speed** | | | | **NART Error Score**  **Intelligence** | | | |  |
| --- | --- | --- | --- | --- | --- | --- | --- | --- | --- | --- | --- | --- | --- | --- | --- | --- | --- | --- | --- | --- | --- | --- | --- | --- | --- | --- | --- | --- |
|  | **OR 95% CI (P-Value)** | | | | **OR 95% CI (P-Value)** | | **OR 95% CI (P-Value)** | | | **OR 95% CI (P-Value)** | | | | | **OR 95% CI (P-Value)** | | | | | **OR 95% CI**  **(P-Value)** | | | | **OR 95% CI**  **(P-Value)** | | | |  |
| N (bottom 10thPCTILE) <65 Yrs.  N (in bottom 10thPCTILE) ≥ 65 Yrs. | | | | 3071 (199)  5137 (832) | 2997 (142)    4820 (857) | | 2754 (154)  4282 (670) | | | 3057 (170)  5081 (702) | | | | | 3055 (330)  5078 (1185) | | | | | 2658 (162)  4244 (520) | | | | 2975 (260)  4871 (546) | | | |  |
| Age (<65 Yrs.)  per 5 year increase | | 1.46 | | 1.16, 1.86  (p=0.02) | 1.74 | 1.29, 2.35  (P<0.001) | 1.53 | 1.16, 2.00  (0.002) | | 1.24 | | 0.80, 1.56  (0.08) | | | 1.31 | 1.10, 1.56  (0.003) | | | | 0.9 | | 0.75, 1.15  (0.5) | | 0.99 | | 0.82, 1.20  (0.9) | |  |
| (≥ 65 Yrs.) | | 1.44 | | 1.34, 1.54 | 1.69 | 1.58, 1.82 | 1.52 | 1.41, 1.64 | | 1.37 | | 1.28, 1.47 | | | 1.39 | 1.31, 1.48 | | | | 1.52 | | 1.40, 1.66 | | 0.88 | | 0.80, 0.96 | |  |
|  | |  | | (P<0.001) |  | (P<0.001) |  | (P<0.001) | |  | | (P<0.001) | | |  | (P<0.001) | | | |  | | (P<0.001) | |  | | (0.03) | |  |
| Sex (<65 Yrs.)  Men vs Women ^a^ | | 1.28 | | 0.95, 1.73  (0.8) | 2.13 | 1.50, 3.02 (P<0.001) | 1.36 | 0.97, 1.90  (0.2) | | 1.79 | | 1.30, 2.45 (P<0.001) | | | 1.59 | 1.26, 2.01 (P<0.001) | | | | 0.99 | | 0.71, 1.37  (0.9) | | 2.00 | | 1.51, 2.63  (P<0.001) | |  |
| (≥ 65 Yrs.) | | 1.02 | | 0.87, 1.20 | 1.96 | 1.66, 2.31 | 1.11 | 0.93, 1.33 | | 1.57 | | 1.33, 1.87 | | | 1.41 | 1.23, 1.62 | | | | 1.23 | | 1.01, 1.50 | | 1.68 | | 1.37, 2.04 | |  |
|  | |  | | (0.1) |  | (P<0.001) |  | (0.08) | |  | | (P<0.001) | | |  | (P<0.001) | | | |  | | (0.04) | |  | (P<0.001) | | |  |
| Marital st.(<65 Yrs.)  Single vs Married ^a^ | | 1.14 | | 0.76, 1.70  (0.5) | 1.38 | 0.87, 2.18  (0.2) | 1.78 | 1.19, 2.67  (0.01) | | 1.11 | | 0.72, 1.71  (0.08) | | | 1.17 | 0.85, , 1.61  (0.3) | | | | 0.74 | | 0.45, 1.21  (0.2) | | 1.14 | | 0.79, 1.66  (0.5) | |  |
| (≥ 65 Yrs.) | | 1.06 | | 0.89, 1.27  (0.5) | 0.95 | 0.78, 1.15  (0.6) | 1.15 | 0.94, 1.40  (0.2) | | 1.40 | | 1.16, 1.69  (0.001) | | | 1.08 | 0.92, 1.26  (0.4) | | | | 1.08 | | 0.86, 1.35  (0.5) | | 1.03 | | 0.81, 1.31  (0.8) | |  |
| Social Class (<65 Yrs.) Manual vs  Non- Manual ^a^ (≥ 65 Yrs.) | | 1.71 | | 1.25, 2.34  (0.001) | 1.14 | 0.79, 1.64  (0.5) | 1.44 | 1.01, 2.05 (0.05) | | 1.45 | | 1.03, 2.03  (0.03) | | | 1.41 | 1.09, 1.81  (0.01) | | | | 1.17 | | 0.83, 1.66  (0.4) | | 1.91 | | 1.44, 2.54  (P<0.001) | |  |
|  | | 1.67 | | 1.42, 1.96  (P<0.001) | 1.60 | 1.35, 1.89  (P<0.001) | 1.18 | 0.98, 1.43  (0.08) | | 1.42 | | 1.19, 1.69  (P<0.001) | | | 1.20 | 1.04, 1.39  (0.02) | | | | 1.10 | | 0.89, 1.35  (0.4) | | 3.01 | | 2.46, 3.67  (P<0.001) | |  |
| Education | | |  |  |  |  |  | |  | | |  | |  |  | | | |  | | |  |  |  | | |  | |
| Age 16 or 18 vs None ^a^ | | |  |  |  |  |  | |  | | |  | |  |  | | | |  | | |  |  |  | | |  | |
| (<65 Yrs.) | | 0.46 | | 0.33, 0.63  (P<0.001) | 0.34 | 0.23, 0.50  (P<0.001) | 0.44 | 0.30, 0.63  (P<0.001) | | 0.71 | 0.49, 1.04  (0.08) | | | | 0.71 | | 0.54, 0.95  (0.02) | | | 0.73 | | 0.49, 1.08  (0.1) | | 0.21 | | 0.16, 0.28  (P<0.001) | |  |
| (≥ 65 Yrs.) | | 0.51 | | 0.43, 0.60  (P<0.001) | 0.66 | 0.55, 0.78  (P<0.001) | 0.72 | 0.60, 8.87  (0.01) | | 0.73 | | | 0.61, 0.87  (0.001) | | 0.74 | | 0.64, 0.86  (P<0.001) | | | 0.75 | | 0.60, 0.92  (0.01) | | 0.31 | | 0.25, 0.37  P<0.001 | |  |
| Graduate level vs None^a^ (<65 Yrs.) | | 0.20 | | 0.11, 0.36  (P<0.001) | 0.14 | 0.07, 0.28  (P<0.001) | 0.30 | 0.16, 0.54  (P<0.001) | | 0.52 | 0.30, 0.91  (0.02) | | | | 0.50 | | | 0.33, 0.76  (0.001) | | | 0.62 | 0.36, 1.07  (0.09) | | 0.05 | | 0.02, 0.10  (P<0.001) | |  |
| (≥ 65 Yrs.) | | 0.27 | | 0.19, 0.36  (P<0.001) | 0.29 | 0.22, 0.40 (P<0.001) | 0.47 | 0.35, 0.64 (P<0.001) | | 0.54 | | | 0.40, 0.72 (P<0.001) | | 0.59 | | | 0.47, 0.74 (P<0.001) | | | 0.69 | 0.50, 0.94  (0.02) | | 0.04 | | 0.02,0.09 (P<0.001) | |  |

^a^ Reference category

Odds ratios for poor performance (defined as obtaining a score less than a cut-off point corresponding to the 10th Percentile of the population distribution adjusted for covariates (age, sex, marital Status, social class and education) stratified by those under the age of 65 years and those 65 years and older.

Abbreviations: A Level, Advanced Level; CANTAB-PAL, Cambridge Neuropsychological Test Automated Battery Paired Associates Learning Test; CI, Confidence Interval; Ed, Education; FTMS, First Trial Memory Score; Grad, graduate; HVLT, Hopkins Verbal Learning Test; NART, National Adult Reading Test; N, Number; O Level, Ordinary Level; OR, Odds ratio; PCTILE, Percentile; Rxn, Reaction; SF-EMSE:, Shortened version (Short form) of the Extended Mental State Exam; SD, Standard deviation; st, status; VST, Visual Sensitivity Test; Yrs., Years
