# Supplementary material for: Cross Sectional Associations between Socio-Demographic Factors and Cognitive Performance in an Older British Population: The European Investigation of Cancer in Norfolk (EPIC-Norfolk) Study
Source: PLoS One. 2016 Dec 8;11(12):e0166779. doi: 10.1371/journal.pone.0166779 (PMC5145160; doi:10.1371/journal.pone.0166779)
Supplement: S3 Table — (DOCX) [file pone.0166779.s003.docx]

Supplementary Table S3. Odds ratios for poor performance using two separate measures of social class for women.

|  | **SF-EMSE**  **Global function** | | | **HVLT**  **Verbal episodic memory** | | | **FTMS**  **Non-verbal episodic memory** | | **PW-Accuracy** | | | **Prospective Memory** | | | **VST Rxn Time**  **Processing Speed** | | **NART Error Score**  **Intelligence** | |
| --- | --- | --- | --- | --- | --- | --- | --- | --- | --- | --- | --- | --- | --- | --- | --- | --- | --- | --- |
|  | OR | | 95% CI  (p value) | OR | | 95% CI  (p value) | OR | 95% CI  (p value) | OR | | 95% CI  (p value) | OR | | 95% CI  (p value) | OR | 95% CI  (p value) | OR | 95% CI  (p value) |
|  |  | | |  | | |  | |  | | |  | | |  | |  | |
| Number of Participants | 8208 | | | 7817 | | | 7036 | | 8138 | | | 8403 | | | 6902 | | 7846 | |
| **Based on partner (Manual vs Non- Manual ^a^) | 1.68 | 1.45, 1.94  (*P*<0.001) | | 1.52 | 1.31, 1.77  (*P*<0.001) | | 1.24 | 1.06, 1.46  (*P*=0.01) | 1.42 | 1.22, 1.66  (*P*<0.001) | | 1.25 | 1.10, 1.42  (*P*=0.001) | | 1.10 | 0.92, 1.31  (*P*=0.3) | 2.63 | 2.23, 3.09  (*P*<0.001) |
| Number of Participants | 8043 | | | 7659 | | | 6889 | | 7976 | | | 7970 | | | 6760 | | 7689 | |
| Based on own social class  (Manual vs Non- Manual ^a^) | 1.98 | 1.70, 2.30  (*P*<0.001) | | 1.69 | 1.45,1.98  (*P*<0.001) | | 1.44 | 1.22, 1.71  (*P*<0.001) | 1.46 | 1.24, 1.72  (*P*<0.001) | | 1.50 | 1.31, 1.71  (*P*<0.001) | | 1.05 | 0.87, 1.27  (*P*=0.6) | 2.97 | 2.52, 3.50  (*P*<0.001) |

** As presented in Table 5

^a^ Reference category

Odds ratios for poor performance (defined as obtaining a score less than a cut-off point corresponding to the 10th Percentile of the population distribution adjusted for all covariates using ‘conventional’ method using partner's occupation/social class for women in analysis (as presented in Table 3) and individuals’ own occupation/social class.

Abbreviations: A Level, Advanced Level; CANTAB-PAL, Cambridge Neuropsychological Test Automated Battery Paired Associates Learning Test; CI, Confidence Interval, FTMS, First Trial Memory Score; HVLT, Hopkins Verbal Learning Test; NART, National Adult Reading Test; N, Number; O Level, Ordinary Level; OR, Odds ratio, Rxn, Reaction; SF-EMSE:, Shortened version (Short form) of the Extended Mental State Exam; SD, Standard deviation; VST, Visual Sensitivity Test
