# Supplementary material for: Cross Sectional Associations between Socio-Demographic Factors and Cognitive Performance in an Older British Population: The European Investigation of Cancer in Norfolk (EPIC-Norfolk) Study
Source: PLoS One. 2016 Dec 8;11(12):e0166779. doi: 10.1371/journal.pone.0166779 (PMC5145160; doi:10.1371/journal.pone.0166779)
Supplement: S4 Table — (DOCX) [file pone.0166779.s004.docx]

Table S4. Odds ratios for poor performance adjusted for all covariates plus NART Error Score

|  | **SF-EMSE**  **Global function** | | | **HVLT**  **Verbal episodic memory** | | | **FTMS**  **Non-verbal episodic memory** | | | **PW-Accuracy**  **Attention** | | | **Prospective**  **Memory** | | | **VST (Rxn. Time)**  **Processing Speed** | | |
| --- | --- | --- | --- | --- | --- | --- | --- | --- | --- | --- | --- | --- | --- | --- | --- | --- | --- | --- |
|  | OR | 95% CI  (p value) | | OR | | 95% CI  (p value) | OR | 95% CI  (p value) | | OR | 95% CI  (p value) | | OR | 95% CI  (p value) | | OR | 95% CI  (p value) | |
| Number of Participants included in analysis | 7778 | | | 7598 | | | 6711 | | | 7742 | | | 7731 | | | 6714 | | |
| Age (per 5 year increase) | 1.51 | | 1.43, 1.59 (*P*<0.001) | 1.75 | 1.66, 1.84 (*P*<0.001) | | 1.51 | | 1.43, 1.59 (*P*<0.001) | 1.41 | | 1.34, 1.48 (*P*<0.001) | 1.39 | | 1.34, 1.45 (*P*<0.001) | 1.38 | | 1.31, 1.46 (*P*<0.001) |
| Sex (Men vs Women ^a^) | 0.83 | | 0.71, 0.97  (*P*=0.02) | 1.78 | 1.52, 2.07 (*P*<0.001) | | 1.08 | | 0.92, 1.27  (*P*=0.4) | 1.52 | | 1.29, 1.78 (*P*<0.001) | 1.37 | | 1.21, 1.55 (*P*<0.001) | 1.14 | | 0.96, 1.36  (*P*=0.1) |
|  |  | |  |  |  | |  | |  |  | |  |  | |  |  | |  |
| Marital status  (Single vs Married ^a^) | 1.02 | | 0.85, 1.22  (*P*=0.9) | 1.00 | 0.83, 1.20 (P=1.00) | | 1.23 | | 1.02, 1.49  (*P*= 0.03) | 1.31 | | 1.09, 1.57 (*P*=0.004) | 1.10 | | 0.95, 1.28 (*P*=0.2) | 1.03 | | 0.84, 1.26 (*P*=0.8) |
|  |  | |  |  |  | |  | |  |  | |  |  | |  |  | |  |
|  |  | |  |  |  | |  | |  |  | |  |  | |  |  | |  |
| Social Class  (Manual vs Non- Manual ^a^) | 1.19 | | 1.02, 1.40  (*P*=0.03) | 1.17 | 0.99, 1.37 (*P*=0.06) | | 1.05 | | 0.88, 1.25  (*P*=0.6) | 1.12 | | 0.94, 132  (*P*=0.2) | 1.09 | | 0.95, 1.24  (*P*=0.2) | 1.12 | | 0.92, 1.34  (*P*=0.3) |
|  |  | |  |  |  | |  | |  |  | |  |  | |  |  | |  |
|  |  | |  |  |  | |  | |  |  | |  |  | |  |  | |  |
| Education |  | |  |  |  | |  | |  |  | |  |  | |  |  | |  |
| Age 16 or 18 vs No Qualifications ^a^ | 0.77 | | 0.65, 0.91  (*P*=0.02) | 0.87 | 0.73, 1.03  (*P*=0.1) | | 0.82 | | 0.68, 0.98  (*P*=0.03) | 0.97 | | 0.81, 1.16  (*P*=0.7) | 0.89 | | 0.77, 1.02  (*P*=0.1) | 0.79 | | 0.64, 0.97  (*P*=0.02) |
|  |  | |  |  |  | |  | |  |  | |  |  | |  |  | |  |
| Graduate level vs No Qualifications ^a^ | 0.70 | | 0.51, 0.96  (*P*=0.03) | 0.54 | 0.39, 0.74  (*P*<0.001) | | 0.66 | | 0.49, 0.90 (*P*=0.01) | 0.97 | | 0.72, 1.29 (*P*=0.8) | 0.83 | | 0.66, 1.04  (*P*=0.10) | 0.79 | | 0.58, 1.07 (*P*=0.1) |
| NART Error Score | 1.07 | | 1.06, 1.08  (*P*<0.001) | 1.06 | 1.05, 1.07  (*P*<0.001) | | 1.04 | | 1.02, 1.04  (*P*<0.001) | 1.05 | | 1.04, 1.04 (*P*<0.001) | 1.03 | | 1.02, 1.04  (*P*<0.001) | 1.01 | | 1.00, 1.02 (*P*=0.3) |

^a^ Reference category

Odds Ratios for poor performance (defined as obtaining a score less than a cut-off point corresponding to the 10th Percentile of the population distribution) for each test in the cognition battery used in EPIC-Norfolk 3, adjusted for covariates, age, sex, marital status, social class, education and NART Error Score.

Abbreviations: A Level, Advanced Level; CANTAB-PAL, Cambridge Neuropsychological Test Automated Battery Paired Associates Learning Test; CI, Confidence Interval, FTMS, First Trial Memory Score; HVLT, Hopkins Verbal Learning Test; NART, National Adult Reading Test; N, Number; O Level, Ordinary Level; OR, Odds ratio, Rxn, Reaction; SF-EMSE:, Shortened version (Short form) of the Extended Mental State Exam; SD, Standard deviation; VST, Visual Sensitivity Test
