# Supplementary material for: Cross Sectional Associations between Socio-Demographic Factors and Cognitive Performance in an Older British Population: The European Investigation of Cancer in Norfolk (EPIC-Norfolk) Study
Source: PLoS One. 2016 Dec 8;11(12):e0166779. doi: 10.1371/journal.pone.0166779 (PMC5145160; doi:10.1371/journal.pone.0166779)
Supplement: S5 Table — (DOCX) [file pone.0166779.s005.docx]

Table S5. Odds ratios for poor performance for participants with complete data (N=5727).

|  | **SF-EMSE** | | **HVLT** | | **FTMS** | | **PW-Accuracy** | | **Prospective Memory** | | **VST Rxn. Time** | | **NART Error Score** | |
| --- | --- | --- | --- | --- | --- | --- | --- | --- | --- | --- | --- | --- | --- | --- |
|  | **Global function** | | **Verbal episodic memory** | | **Non-verbal episodic memory** | | **Attention** | |  |  | **Processing speed** | | **Intelligence** | |
|  | **OR** | **95% CI (P-value)** | **OR** | **95% CI**  **(P-value)** | **OR** | **95% CI**  **(P-value)** | **OR** | **95% CI (P-value)** | **OR** | **95% CI (P-value)** | **OR** | **95% CI (P-value)** | **OR** | **95% CI (P-value)** |
| Age (per 5 year increase) | 1.43 | 1.34, 1.52 | 1.65 | 1.55, 1.76 | 1.49 | 1.40, 1.58 | 1.37 | 1.29, 1.45 | 1.40 | 1.33, 1.45 | 1.34 | 1.26, 1.42 | 1.00 | 0.90, 1.02 |
|  |  | ('P<0.001) |  | (P<0.001) |  | (P<0.001) |  | (P<0.001) |  | (P<0.001) |  | (P<0.001) |  | (0.2) |
| Sex (Men vs Women*) | 0.93 | 0.78, 1.11 | 1.94 | 1.62, 2.32 | 1.16 | 0.97, 1.39 | 1.63 | 1.35, 1.96 | 1.39 | 1.21, 1.61 | 1.18 | 0.97, 1.42 | 1.71 | 1.42, 2.07 |
|  |  | (0.5) |  | (P<0.001) |  | (0.1) |  | (P<0.001) |  | (P<0.001) |  | (0.09) |  | (P<0.001) |
| Marital status  (Single vs Married ^a^) | 1.04 | 0.83, 1.29 | 0.95 | 0.76, 1.18 | 1.16 | 0.94, 1.44 | 1.34 | 1.08, 1.66 | 1.03 | 0.86, 1.22 | 1.09 | 0.87, 1.37 | 0.95 | 0.74, 1.21 |
|  |  | (0.7) |  | (0.6) |  | (0.2) |  | (0.01) |  | (0.8) |  | (0.4) |  | (0.7) |
| Social Class  (Manual vs Non- Manual ^a^) | 1.65 | 1.37, 1.98 | 1.56 | 1.30, 1.87 | 1.26 | 1.05, 1.52 | 1.44 | 1.19, 1.75 | 1.21 | 1.04, 1.41 | 1.13 | 0.92, 1.38 | 2.59 | 2.14, 3.15 |
|  |  | (P<0.001 |  | (P<0.001) |  | (0.02) |  | (P<0.001) |  | (0.01) |  | (0.3) |  | (P<0.001) |
| Education |  |  |  |  |  |  |  |  |  |  |  |  |  |  |
| Age 16 or 18 vs No Qualifications ^a^ | 0.52 | 0.43, 0.62 | 0.62 | 0.51, 0.75 | 0.65 | 0.53, 0.78 | 0.69 | 0.57, 0.85 | 0.72 | 0.61, 0.85 | 0.77 | 0.62, 0.95 | 0.26 | 0.22, 0.32 |
|  |  | (P<0.001) |  | (P<0.001) |  | (P<0.001) |  | (P<0.001) |  | (P<0.001) |  | (0.02) |  | (P<0.001) |
| Graduate level vs No Qualifications ^a^ | 0.23 | 0.16, 0.33 | 0.23 | 0.16, 0.34 | 0.35 | 0.26, 0.50 | 0.50 | 0.36, 0.69 | 0.54 | 0.42, 0.68 | 0.74 | 0.54, 1.003 | 0.04 | 0.02, 0.07 |
|  |  | (P<0.001) |  | (P<0.001) |  | (P<0.001) |  | (P<0.001) |  | (P<0.001) |  | (0.05) |  | (P<0.001) |

^a^ Reference category

Sensitivity analysis with Odds ratios for poor performance (defined as obtaining a score less than a cut-off point corresponding to the 10th Percentile of the population distribution adjusted for covariates (age, sex, marital Status, social class and education) for the 5727 participants with complete data on all seven cognitive tests.

Abbreviations: A Level, Advanced Level; CANTAB-PAL, Cambridge Neuropsychological Test Automated Battery Paired Associates Learning Test; CI, Confidence Interval, FTMS, First Trial Memory Score; HVLT, Hopkins Verbal Learning Test; NART, National Adult Reading Test; N, Number; O Level, Ordinary Level; OR, Odds ratio, Rxn, Reaction; SF-EMSE:, Shortened version (Short form) of the Extended Mental State Exam; SD, Standard deviation; VST, Visual Sensitivity Test
